# Source EEG reveals that Rolandic epilepsy is a regional epileptic encephalopathy

**DOI:** 10.1016/j.nicl.2022.102956

**Published:** 2022-02-07

**Authors:** Elizabeth R. Spencer, Dhinakaran Chinappen, Britt C. Emerton, Amy K. Morgan, Matti S. Hämäläinen, Dara S. Manoach, Uri T. Eden, Mark A. Kramer, Catherine J. Chu

**Affiliations:** a Graduate Program in Neuroscience, Boston University, Boston, MA 02215; b Department of Neurology, Massachusetts General Hospital, Boston, MA 02114; c Department of Psychiatry, Massachusetts General Hospital, Boston, MA 02114; d Harvard Medical School, Boston, MA 02115; e Athinoula A. Martinos Center for Biomedical Imaging, Charlestown, MA 02129; f Massachusetts General Hospital, Department of Radiology, Boston, MA 02114; g Department of Mathematics and Statistics, Boston University, Boston, MA 02215; h Center for Systems Neuroscience, Boston University, Boston, MA 02215

**Keywords:** CTOPP-2, Comprehensive Test of Phonological Processing, 2nd ed, ESI, Electrical source imaging, FDR, False discovery rate, GPB, Grooved Pegboard, MEMPRAGE, Multi‐echo magnetization‐prepared rapid acquisition gradient‐echo, NREM, Non-rapid eye movement, PSI, Processing Speed Index, PV, Parvalbumin, SOM, Somatostatin, TRN, Thalamic reticular nucleus, WISC-V, Wechsler Intelligence Scale for Children, 5th ed, Childhood epilepsy with centrotemporal spikes, Epileptic encephalopathy, Electrical source imaging, Thalamus, Sleep spindle, EEG

## Abstract

Rolandic epilepsy is the most common form of epileptic encephalopathy, characterized by sleep-potentiated inferior Rolandic epileptiform spikes, seizures, and cognitive deficits in school-age children that spontaneously resolve by adolescence. We recently identified a paucity of sleep spindles, physiological thalamocortical rhythms associated with sleep-dependent learning, in the Rolandic cortex during the active phase of this disease. Because spindles are generated in the thalamus and amplified through regional thalamocortical circuits, we hypothesized that: 1) deficits in spindle rate would involve but extend beyond the inferior Rolandic cortex in active epilepsy and 2) regional spindle deficits would better predict cognitive function than inferior Rolandic spindle deficits alone. To test these hypotheses, we obtained high-resolution MRI, high-density EEG recordings, and focused neuropsychological assessments in children with Rolandic epilepsy during active (*n* = 8, age 9–14.7 years, 3F) and resolved (seizure free for > 1 year, *n* = 10, age 10.3–16.7 years, 1F) stages of disease and age-matched controls (*n* = 8, age 8.9–14.5 years, 5F). Using a validated spindle detector applied to estimates of electrical source activity in 31 cortical regions, including the inferior Rolandic cortex, during stages 2 and 3 of non-rapid eye movement sleep, we compared spindle rates in each cortical region across groups. Among detected spindles, we compared spindle features (power, duration, coherence, bilateral synchrony) between groups. We then used regression models to examine the relationship between spindle rate and cognitive function (fine motor dexterity, phonological processing, attention, and intelligence, and a global measure of all functions). We found that spindle rate was reduced in the inferior Rolandic cortices in active but not resolved disease (active *P* = 0.007*;* resolved *P* = 0.2) compared to controls. Spindles in this region were less synchronous between hemispheres in the active group (*P* = 0.005; resolved *P* = 0.1) compared to controls; but there were no differences in spindle power, duration, or coherence between groups. Compared to controls, spindle rate in the active group was also reduced in the prefrontal, insular, superior temporal, and posterior parietal regions (*i.e.,* “regional spindle rate”, *P* < 0.039 for all). Independent of group, regional spindle rate positively correlated with fine motor dexterity (*P* < 1e-3), attention (*P* = 0.02), intelligence (*P* = 0.04), and global cognitive performance (*P* < 1e-4). Compared to the inferior Rolandic spindle rate alone, models including regional spindle rate trended to improve prediction of global cognitive performance (*P* = 0.052), and markedly improved prediction of fine motor dexterity (*P* = 0.006). These results identify a spindle disruption in Rolandic epilepsy that extends beyond the epileptic cortex and a potential mechanistic explanation for the broad cognitive deficits that can be observed in this epileptic encephalopathy.

## Highlights

• Children with RE have fewer spindles but they have typical time–frequency features.
• Spindle deficits extend to multiple cortical regions in Rolandic epilepsy.
• Cognitive deficits are predicted by spindle rate in Rolandic epilepsy.
• Regional spindle rate predicts motor deficits better than Rolandic spindle deficit.
• Spindle features in RE identify a regional thalamocortical epileptic encephalopathy.

## Graphical abstract

**Figure:**
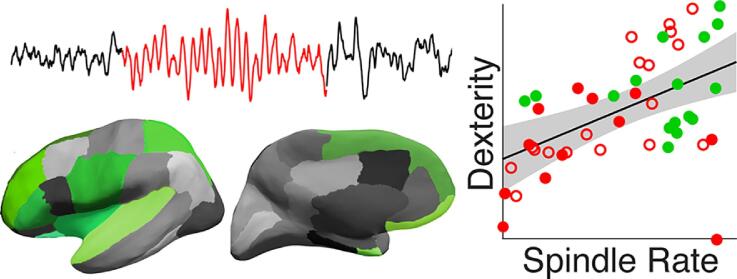

## 1 Introduction

Rolandic epilepsy, previously known as benign epilepsy with centrotemporal spikes, is the most common form of childhood epileptic encephalopathy, characterized by epileptic spikes and seizures arising from the inferior Rolandic cortex during non-rapid eye movement (NREM) sleep and neurocognitive deficits ranging from subtle to severe in school-age children (Carvill et al., 2013, Berg et al., 2010, Tovia et al., 2011, Besenyei et al., 2012). Common cognitive deficits grossly localize to frontoparietal and temporal cortical processes and include sensorimotor dysfunction, attention-regulation difficulties, and phonological processing difficulties (Callenbach et al., 2010, Katewa and Parakh, 2015, Vannest et al., 2015, Wickens et al., 2017). This disease is self-limited, in that by adolescence, seizures spontaneously resolve. Additionally, the neurocognitive deficits are also transient, where formal neuropsychological testing identifies symptoms in most children tested within approximately five years of onset (Wickens et al., 2017) but cannot detect deficits when children are tested nine years after diagnosis (Ross et al., 2020). Importantly, cognitive deficits are observed in children independent of anticonvulsant treatment status (Wickens et al., 2017).

While epileptic spikes provide a robust biomarker of seizure risk, the neurophysiological basis for cognitive deficits in this epileptic encephalopathy remains largely unknown. The activation of epileptic spikes during NREM sleep characteristic of Rolandic epilepsy (Carvill et al., 2013, Katewa and Parakh, 2015) suggest involvement of the thalamus, a prominent brain nucleus involved in synchronizing and regulating sleep rhythms (De Gennaro and Ferrara, 2003, Gent et al., 2018). This hypothesis has been further supported by recent work identifying abnormal thalamocortical white matter connectivity to the Rolandic cortex (Thorn et al., 2020) and a paucity of sleep spindles, characteristic 10–15 Hz (sigma band) oscillations produced during NREM sleep, in central regions on scalp EEG (Kramer et al., 2021). Sleep spindles are generated and amplified within the thalamocortical circuits and have been associated with sleep-dependent memory consolidation, and general cognitive functioning (De Gennaro and Ferrara, 2003, Beenhakker and Huguenard, 2009). Epileptiform spikes are anticorrelated with spindles in Rolandic epilepsy (Kramer et al., 2021), suggesting a competitive relationship, whereby spikes may hijack and disrupt spindle thalamocortical circuitry (Beenhakker and Huguenard, 2009).

The thalamus is comprised of nuclei with far reaching and distinct cortical projections (Behrens et al., 2003, Fama and Sullivan, 2015, Bastuji et al., 2020). Recent evidence reveals that spindles can be initiated by focal generators in the thalamus (Bastuji et al., 2020) and it has long been known that distinct thalamic nuclei project spindles to distinct regional cortical areas (Andersen et al., 1967). Thalamocortical circuit dysfunction in epilepsy may therefore result in spindle deficits that extend beyond the focal epileptiform cortical source. Although in Rolandic epilepsy the epileptiform spikes have been well-localized to the inferior Rolandic cortices (Ross et al., 2020, Mirandola et al., 2013), the spatial extent of the spindle disruption is not known. The impact of a regional spindle deficit compared to a focal spindle deficit on cognitive function is not known. However, since focal spindle-related reactivations can support performance on specific cognitive tasks (Bergmann et al., 2012, Cowan et al., 2020), a regional spindle deficit would be expected to have broader consequences on cognitive functions than a focal spindle deficit.

Prior work evaluating sleep spindles in Rolandic epilepsy utilized standard scalp EEG data, which is limited in spatial resolution due to skull blurring and inconsistent electrode placement across subjects (Kramer et al., 2021). To better evaluate the spatial extent of the spindle deficit in Rolandic epilepsy, we utilized co-registered high-density EEG, high-resolution MRI, digitized electrode coordinates, and a validated biophysical electrical source imaging (ESI) model to estimate cortical signals (Hamalainen and Sarvas, 1987, Hamalainen and Sarvas, 1989). To relate these findings to cognitive function, we evaluated performance on tasks targeting sensorimotor, attention, phonological processing, and global intellectual (IQ) skills, the canonical challenges reported in Rolandic epilepsy (Vannest et al., 2015, Wickens et al., 2017, Scheffer et al., 2017). We hypothesized that: 1) spindle rate would be decreased in the inferior Rolandic cortex, but that spindle deficits would also extend beyond the epileptic cortex, and 2) regional spindle rates estimated from all regions exhibiting a spindle deficit would better predict cognitive function than focal spindle rates estimated solely from the inferior Rolandic cortex. Identification of regional spindle deficits in Rolandic epilepsy may provide an improved biomarker and mechanistic explanation for the variable cognitive deficits observed in children with this epileptic encephalopathy and evidence for a regional disruption to the thalamocortical circuit in this disease.

## 2 Materials and methods

We tested two a priori hypotheses: 1) spindle rate would be decreased in the inferior Rolandic cortex and in regions beyond the epileptic cortex in active epilepsy; and 2) spindle rates estimated from all regions exhibiting a spindle deficit would better predict cognitive function than focal spindle rates estimated solely from the inferior Rolandic cortex. For hypothesis (1), we tested whether spindles or spectral estimates that approximate spindle activity (*e.g.*, sigma power) were focally reduced in the inferior Rolandic cortex in epilepsy groups as compared to a control group. We then tested if the identified focal spindles were different between epilepsy and control groups (*i.e.*, power, duration, coherence, synchrony). Finally, we tested if spindles were regionally reduced in epilepsy groups compared to a control group beyond the inferior Rolandic cortex. For hypothesis (2), we first tested if spindle rate predicted cognitive function by building models relating focal and regional estimates of spindle rate to performance on four neuropsychological assessments. Then, we tested if the models using regional spindle rate improved prediction compared to models using focal spindle rate. We analyzed both a global model of cognitive function combining performance on all assessments as well as each of the four neuropsychological assessments individually.

### 2.1 Subject data

Children with a documented EEG showing sleep-activated centrotemporal spikes and a clinical diagnosis of Rolandic epilepsy by a child neurologist (*n* = 18, age 9–16.7, 4F) and control subjects (*n* = 8, age 8.9–14.5 years, 5F) were recruited to participate. The epilepsy subjects were further divided into two groups based on seizure risk (Ross et al., 2020): active disease (*n* = 8, age 9–14.7 years, 3F), and resolved disease, defined as seizure-free for at least 12 months after which the majority of subjects have no further seizures (*n* = 10, age 10.3–16.7 years, 1F).

This research was approved by the Massachusetts General Hospital and Boston University institutional review boards, and assent and informed consent were obtained from each subject and guardian.

### 2.2 Neuropsychological assessment

Each subject completed a focused neuropsychological assessment performed by clinical neuropsychologists (BCE, AKM) including standardized tests of fine motor dexterity, processing speed, global intellectual function, and phonological awareness. To test fine motor dexterity, subjects completed the Grooved Pegboard (GPB) task, where the time required to correctly place grooved pegs into notched holes at different orientations is recorded, thereby providing an assessment of hand-eye coordination, motor speed and sensorimotor control and integration (Merker et al., 2018). Subjects (active epilepsy *n* = 6; resolved epilepsy *n* = 9*;* control *n* = 8) completed the GPB task once using their dominant hand and once using their nondominant hand (Fig. 7A). Processing speed was assessed using the Processing Speed Index (PSI) from the Wechsler Intelligence Scale for Children, 5th ed (WISC-V), which is derived from subtests that require children to attend to visual material and sort or classify targets and symbols in a time-limited setting. As such, there are demands for sensory registration and timing of motor response (Jacobson et al., 2011) (active epilepsy *n* = 7; resolved epilepsy *n* = 9; control *n* = 8). Global intellectual functioning was estimated using the WISC-V to quantify full-scale IQ, which is derived from subtests of verbal comprehension and knowledge base, visuospatial processing, fluid reasoning, working memory, and processing speed (Wechsler, 2014) (active epilepsy *n* = 6; resolved epilepsy *n* = 8; control *n* = 8). Phonological processing was assessed using Phonological Awareness index from the Comprehensive Test of Phonological Processing, 2nd ed. (CTOPP-2). This index is comprised of three subscales assessing ability to isolate, blend, and otherwise manipulate and recombine phonemes to derive real words (Wagner et al., 1991) (active epilepsy *n* = 5; resolved epilepsy *n* = 7*;* control *n* = 8).

For all tests, z-scores representing each individual’s deviation from standardized score distributions for his or her age were evaluated.

### 2.3 Electrical source imaging and minimum norm estimation

Each subject underwent an EEG recording session, MRI recording session, and neuropsychological evaluation. MRI and neuropsychological evaluations were separated from the EEG by a median of 0 days (range 0–372, interquartile range 1 day) and 23.3 days (range 0–341, interquartile range 41.1 days), respectively. To increase the likelihood of capturing sleep during the EEG recording session, all subjects were given instruction for sleep-deprivation prior to arriving for the EEG recording session following routine procedures for clinical EEGs (recommended maximum of 4 h of sleep the previous night). Subjects were given a nap opportunity for up to 1 h around mid-day. Continuous EEG data (70 channel cap based on the 10–10 electrode placement system with additional electrodes at T1 and T2 (Easycap, Vectorview, Elekta-Neuromag, Helsinki, Finland)) were acquired at a sampling rate of 2035 Hz after bandpass filtering (low pass cutoff frequency of 671.55 Hz). Subsequently, channels with no signal or high noise and periods of artifact were identified through visual analysis by an experienced electroencephalographer and manually removed. EEG data were staged following standard procedures (Grigg-Damberger et al., 2007). Data collected during stages 2 and 3 NREM sleep epochs, when spindles are present and epileptiform spikes are activated, were concatenated and selected for analysis (mean duration 811.9 s, minimum duration 63.7 s, maximum duration 2644.2 s). Although stages 2 and 3 sleep were not distinguished in our analysis, stage 3 sleep was noted to be rare on visual inspection.

MRI data acquisition included T1‐weighted multi‐echo magnetization‐prepared rapid acquisition gradient‐echo (MEMPRAGE) images that were collected on a 3 T MAGNETOM Prisma Scanner (Siemens, Germany) with the following parameters: TR = 2,530 ms, TE = (1.69, 3.55, 5.41, 7.27 ms), voxel size 1x1x1 mm, flip angle = 7 degrees.

Source analysis of EEG data was performed using the MNE-C software package (Hamalainen and Sarvas, 1989, Gramfort et al., 2014). Briefly, MNE provides a distributed source estimate of cortical currents incorporating constraints from the patients' MRI, transforming the data to brain space without requiring heuristic choices or strong assumptions about the sources (Chu et al., 2015).

**Fig. 1:**
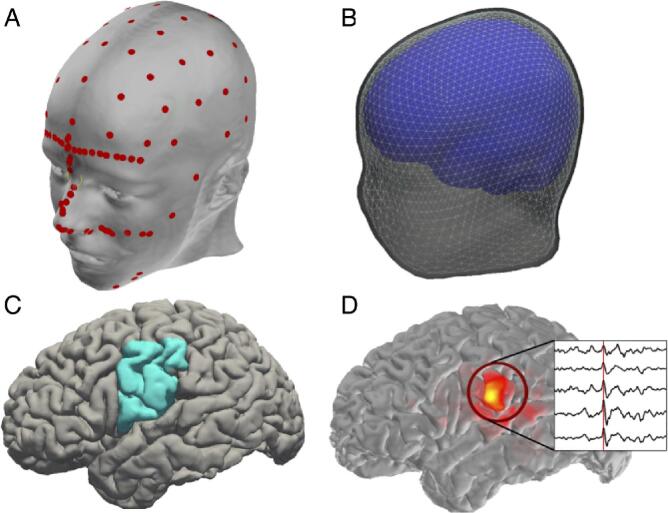
**Example of electrical source imaging procedure.** (**A**) Digitized EEG electrode placement and anatomical landmarks (red circles), and (**B**) reconstructed anatomical surfaces: inner skull (blue), outer skull (light gray), and outer skin surfaces (dark gray). (**C**) Example cortical surface reconstruction with lateral inferior Rolandic cortex indicated (blue). (**D**) Example source activity during an interictal spike in the inferior Rolandic cortex (inset). Red heat-map indicates amplitude of interictal spike (corresponding to time indicated by red line in inset) averaged over time for each source. (For interpretation of the references to colour in this figure legend, the reader is referred to the web version of this article.)

EEG electrode positions were digitized prior to recording using a 3D digitizer (Fastrak, Polhemus Inc., Colchester, VA). Anatomical cortical surfaces of the brain were reconstructed using FreeSurfer from the MEMPRAGE data (Fischl, 2012). Digitized electrode coordinates were aligned to the MEMPRAGE data using the nasion and auricular points as fiducial markers, and random points along the skin surface were also digitized to improve EEG-MRI co-registration (Fig. 1A).

For the forward model, a three-compartment boundary element model bounded by the inner skull, outer skull, and outer skin surfaces with conductivities 0.3 S/m, 1.0 S/m, and 0.006 S/m for scalp, cerebrospinal fluid, and skull, respectively, was generated using the watershed algorithm in FreeSurfer (Fig. 1B). The digitized EEG electrode coordinates were co-registered to the reconstructed surface using the nasion and auricular points. Cortical surfaces were parcellated using FreeSurfer to identify the regions of interest within each subject. A sphere centered on the most inferior vertex in the pre- or post-central gyrus with a radius equal to half of the distance between the most inferior and most superior vertices in the pre- or post-central gyrus was generated individually in each subject. The union of this label and the pre- and post-central gyri labels was used to define the inferior Rolandic cortex label. The overlap between this sphere and the pre- and post-central gyrus labels was the inferior Rolandic cortex ROI (Song et al., 2019) (Fig. 1C).

For each subject, 10,242 source space points per hemisphere were employed in the topology of a recursively subdivided icosahedron. To balance spatial resolution and computational speed in subsequent analysis, we also constructed a lower-density source space with 162 source space points per hemisphere. The inverse operator was computed from the forward solution with a loose orientation constraint of 0.6 to eliminate implausible sources and 2 µV as the estimate of EEG noise. The normal component of dipoles at each source space point were used for source data estimates.

For each subject, we calculated the activity at each of the 162 source space points as follows. First, a circle of approximately 1 cm diameter on the cortical surface was drawn around the source space point using the full-width half-max smoothing kernel (Fischl, 2012). Then, the mean activity of the high-density source space points within this circle was computed; this mean activity defined the average source space solution for the low-density source space point. Example source data estimates during a Rolandic epileptic spike are shown in Fig. 1D.

The source space signals were down-sampled to 407 Hz using MATLAB’s function *decimate*. We restricted our initial analysis to sources in the inferior Rolandic cortices, which are the cortical origins of the epileptic spikes in Rolandic epilepsy subjects. For regional analysis, we evaluated all cortical labels produced using the Desikan-Killiany atlas (Desikan et al., 2006).

### 2.4 Artifact and epileptic spike removal procedures

To minimize the impact of muscle movements, we adapted the artifact removal procedure in Chu *et al.* (Chu et al., 2014). First, for a 1 s interval of data we computed the power spectrum (Hanning taper). Then, we computed a linear fit to the logarithm of power versus logarithm of frequency for frequencies between 30 and 95 Hz. Given the typical $1/f^{\alpha }$ property of EEG activity (He et al., 2010), if the slope of the linear fit was not sufficiently negative, then the interval was marked as an artifact. We chose a threshold ofα = 1.5, which exceeds the values of α typically observed in human brain activity (He et al., 2010).

Large amplitude interictal spikes common in subjects with Rolandic epilepsy produce broadband spectral content and may impact detection and characterization of spindles. Although our spindle detector is robust to the impact of spikes (*e.g.*, see section *Automated spindle detection* and Kramer *et al.* (Kramer et al., 2021), to remove any potential impact of interictal spikes on our source estimates and subsequent analysis, we applied an automated spike detection method - the Persyst 13 algorithm (Scheuer et al., 2017) - to each patient’s scalp EEG data, and identified all spikes at the standard 10–20 EEG channels. We then removed 200 ms around spikes detected on the central, temporal, and frontal electrodes in all subsequent analysis.

### 2.5 Spectral analysis

All spectral analyses were computed using the multitaper method as implemented in the Chronux toolbox (Bokil et al., 2010), unless otherwise noted. For each source, we computed the power spectrum on non-overlapping one second windows (frequency resolution 1 Hz; single Hanning taper) for the entire duration of stages 2 and 3 of NREM sleep, and then averaged these spectra. Then, for each region of interest, in each hemisphere, we averaged the power spectra of all sources within the label to create one power spectrum per label. We normalized this spectrum by the total power between 0 and 50 Hz to compute a relative power spectrum. We then computed two measures of sigma band activity. First, we computed sigma power as the average of relative power between frequencies 10–15 Hz for each label in each hemisphere. Second, we computed the sigma bump (Donoghue et al., 2020, Ouyang et al., September 2019), which we define as the sigma power (10–15 Hz) with background activity subtracted. Specifically, we first fit a line between the power at 10 Hz and 15 Hz to approximate the $1/f^{\alpha }$ spectral background. Then, we subtracted the fit line from the power spectrum, and summed over the positive values between 10 and 15 Hz to approximate the contribution of sigma band activity above the spectral background. We computed this statistic for the inferior Rolandic cortex in both hemispheres.

### 2.6 Automated spindle detection

**Fig. 2:**
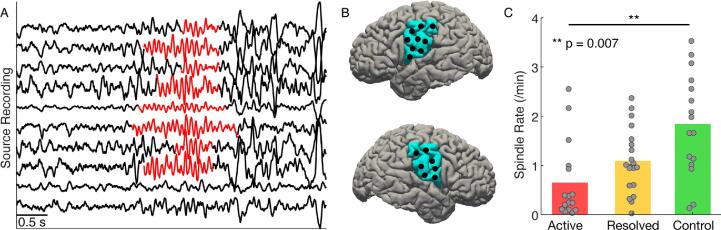
**Spindle rate is lower in subjects with Rolandic epilepsy.** (**A**) Example spindle activity (10–15 Hz rhythms characteristic of NREM sleep) from 10 sources in the left inferior Rolandic cortex with detected spindles (orange). (**B**) Left and right lateral views of the brain with the inferior Rolandic region indicated (blue). Black circles indicate example source locations used to compute spindle rate. (**C**) Spindle rate in the inferior Rolandic cortices for active (red), resolved (yellow), and control (green) subjects. Bar heights indicate population mean, and circles indicate the spindle rate for each hemisphere of each patient. (For interpretation of the references to colour in this figure legend, the reader is referred to the web version of this article.)

Spindles - sigma-band (10–15 Hz) activity of duration 0.5–2 s - are characteristic rhythms present in stages 2 and 3 NREM sleep (examples in Fig. 2A). To identify spindles in subjects with Rolandic epilepsy, we applied a spindle detection method developed to accurately measure sleep spindles in subjects with epilepsy to source activity in the left and right inferior Rolandic cortices (Fig. 2B) of subjects with Rolandic epilepsy and control subjects. (Kramer et al., 2021). Briefly, the method estimates the probability of the spindle state given three features calculated from the source activity: (i) theta power (4–8 Hz), (ii) sigma power (9–15 Hz), and (iii) a measure of the consistency of time intervals between subsequent peaks and subsequent troughs in the signal. This detector was trained and validated using the Rolandic epilepsy and control scalp EEG data; for details see Kramer *et al.* (Kramer et al., 2021). Here, we applied this detector to the activity of each source within a chosen cortical label (*e.g.*, within the left and right inferior Rolandic cortices). The method returns the time interval of each spindle detection, with spindle durations restricted to be at least 0.5 s. We computed the number of spindles over time to define the spindle rate (spindles/minute).

**Fig. 3:**
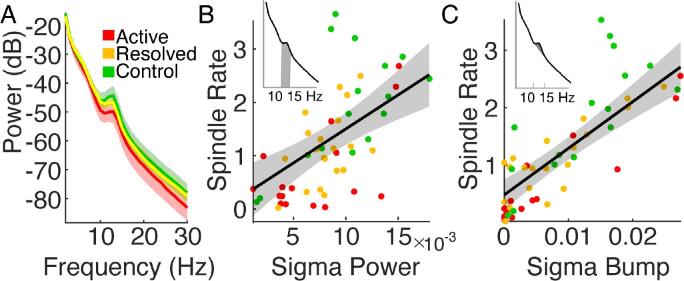
**Spindle rate correlates with other spectral estimates.** (**A**) Averaged power spectra for each patient group, active (red), resolved (yellow), and control subjects (green). Solid lines indicate the mean, and shading indicates 95% confidence intervals. (**B, C**) Spindle rate in the inferior Rolandic cortex correlates with sigma power (**B**) and sigma bump (**C**). Shaded regions in the power spectra insets in the upper left of (**B**) and (**C**) represent areas used to compute sigma power and sigma bump, respectively. Black line indicates the linear fit, shading the 95% confidence intervals, and circles the values for each subject (see legend). (For interpretation of the references to colour in this figure legend, the reader is referred to the web version of this article.)

### 2.7 Assessment of spindle features

We computed five features to characterize the identified spindles: duration, sigma-band power, intra-hemispheric and inter-hemispheric coupling, and bilateral spindle synchrony. We define each feature here.

*Duration*: We measured spindle duration as the time between onset and offset of each spindle detection. As part of the spindle detection procedure, durations were restricted to exceed 0.5 s.

*Sigma-band power*: To compute the sigma-band power of a spindle, we first applied a Hanning window to the source activity during the spindle detection. For spindle durations < 4 s, we then zero padded the signal to 4 s, and evaluated the mean power between 10.25 Hz and 14.75 Hz to span the (De Gennaro and Ferrara, 2003, Behrens et al., 2003) Hz range. We averaged the sigma band power over all spindles from sources within the left (or right) inferior Rolandic cortex for each patient.

**Fig. 4:**
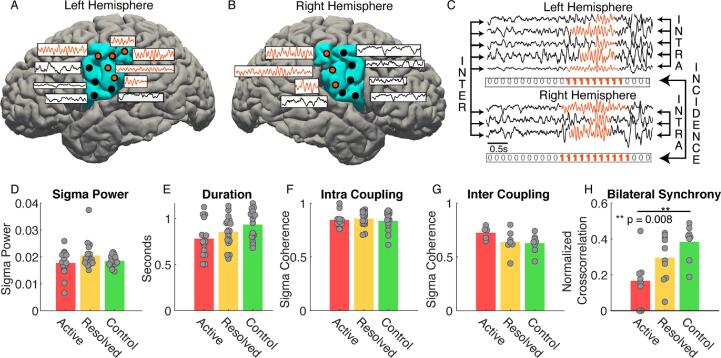
**Rolandic epilepsy subjects produce fewer, but typical, spindles.** (**A**) Left and (**B**) right hemispheres of a subject with sources (circles) in the inferior Rolandic cortices (blue). The subset of sources in the inferior Rolandic cortices with detected spindles are colored orange, otherwise black. (**C**) Example recordings from source in the left hemisphere (top) and the right hemisphere (bottom) with detected spindles in orange. Arrows between sources within each cortex indicate intra-hemispheric coherence, and arrows between sources from the left to the right cortices indicate inter-hemispheric coherence. Below the recordings from each hemisphere is the corresponding spindle indicator function that contains ones if at least one source is exhibiting a spindle at that moment in time and is used to compute the bilateral synchrony of spindles. (**D-H**) Spindle characteristics sigma power (**D**), duration (**E**), intra-hemispheric sigma band coherence **(F)**, inter-hemispheric sigma band coherence **(G)**, and bilateral synchrony **(H)**. We find evidence of a difference between active and control subjects only for the bilateral synchrony (asterisks). (For interpretation of the references to colour in this figure legend, the reader is referred to the web version of this article.)

*Intra-hemispheric and inter-hemispheric sigma-band coherence*: To assess sigma-band coherence, we first identified time intervals of spindle activity. To do so, we defined spindle indicator vectors for the set of sources within the left and right inferior Rolandic cortices. The spindle indicator vector is a time series containing ones when at least one source in a region of interest exhibits a spindle (example indicator vectors computed for sources from the left and right inferior Rolandic gyrus shown in Fig. 4C). We note that, within a spindle indicator vector, multiple sources may be involved, and not all sources may be involved for the entire duration of the vector. We selected ± 1 s around the center of each spindle run to create a 2 s spindle epoch. In these spindle epochs, only sources exhibiting spindles were included to compute the coherence.

Within each spindle epoch, we investigated the coherence of sources within and between the left and right inferior Rolandic cortices. To assess intra-hemispheric coupling, we computed the coherence between sources in the left (or right) inferior Rolandic cortex during each spindle epoch detected in the left (or right) inferior Rolandic cortex, yielding two measurements per subject (see ‘intra’ example in Fig. 4C). To assess inter-hemispheric coupling, we computed coherence between the left and right inferior Rolandic cortices during bilateral and simultaneous spindle epochs detected in both cortices, yielding one measurement per patient (see ‘inter’ example in Fig. 4C).

We computed pairwise coherence between sources with a 2.5 Hz frequency resolution and 9 tapers. We evaluated coherence at 12.5 Hz covering the 12.5 ± 2.5 Hz to estimate the sigma coherence.

*Bilateral synchrony*: Because Rolandic spikes occur independently in the left and right hemispheres in Rolandic epilepsy, and sleep spindles often occur synchronously between hemispheres in the Rolandic regions after ∼ 12 months of age (Gruber and Wise, 2016), we evaluated the interhemispheric synchrony between Rolandic spindles. To do so, we computed the dot product between the spindle indicator vectors for each hemisphere, as defined in the previous section (example indicator vectors in Fig. 4C). The result estimates how often at least one Rolandic cortical source in each hemisphere produce spindles that temporally overlap. To account for potential biases due to differences in spindle rate, we divided the product by the sum of the bilateral spindle run (*i.e.,* a vector that indicates when spindles occurred in either the left or the right hemisphere). For example, in Fig. 4C, we compute the dot product of the left and right indicator vectors (resulting in a value of 9) and divide by the sum of the joint bilateral spindle run (value of 11).

### 2.8 Statistical analysis

To test hypothesis (1), we implemented a mixed effects model with spindle rate as the dependent variable and group as the predictor (indicator vectors for the active group and for the resolved group; both zero if in the control group) and controlling for age. In addition to a direct measure of spindle rate, we also tested two related measures: sigma power and sigma bump (see *Spectral analysis*). We used a linear model fit using maximum likelihood for sigma power (*P* = 0.79, Lilliefors test, no evidence of violation of normality) and a quasi-Poisson model fit using pseudo likelihood for spindle rate and sigma bump. We chose a quasi-Poisson model for the spindle rate data for three reasons. First, visual inspection of the data suggested a concentration of spindle rate values near zero (Fig. 3A). Second, spindle rate is directly related to the (discrete and nonnegative) spindle count, consistent with this discrete probability distribution. Third, we find a near violation of normality (*P* = 0.076, Lilliefors test). We note that, assuming a normal distribution for the spindle rate and repeating all analyses, we found consistent results. We chose a quasi-Poisson model for the sigma bump due to the violation of normality for these data (*P* = 0.01, Lilliefors test) and to maintain consistency with the model of spindle rate. We included a random intercept term to account for repeat measurements (*e.g.*, from the left and right inferior Rolandic cortices) taken from the same subject. Significant differences between the active or resolved epilepsy subjects and control subjects were identified if the p-value of the corresponding variable was < 0.05.

In addition to spindle rate, we tested whether focal spindle properties (*i.e.,* sigma power of spindles, duration, intra-hemispheric coupling, inter-hemispheric coupling, or bilateral synchrony) in the inferior Rolandic cortex differed by group. To do so, we implemented a likelihood ratio test (2 degrees of freedom) comparing a null and a full model. The null model included age, and the full model additionally included two group variables (active and resolved epilepsy). We chose a linear model because we found no violations of normality for any measures (*P* > 0.1, Lilliefors test). For sigma power, duration, and intra-hemispheric coherence, we included a random intercept term because there were two measurements per subject (*e.g.*, from the left and right inferior Rolandic cortices). For inter-hemispheric coherence and bilateral synchrony, we only include the fixed effects because there was only one measurement per subject. We tested for significant differences (*P* < 0.05, chi-squared distribution) between the models using the MATLAB functions, *compare* and *lratiotest*, for the mixed and fixed effects models, respectively. In the full model, we identified significant differences between the active or resolved epilepsy subjects and control subjects if the p-value of the corresponding variable was < 0.05.

To test for spindle deficits outside of the inferior Rolandic cortex, we implemented the same quasi-Poisson mixed effects model for spindle rate used for the inferior Rolandic cortex for each of the 31 Desikan-Killiany Atlas labels. We use false discovery rate (FDR) (Benjamini and Hochberg, 1995) with *q* = 0.05 to correct for multiple comparisons.

**Fig. 5:**
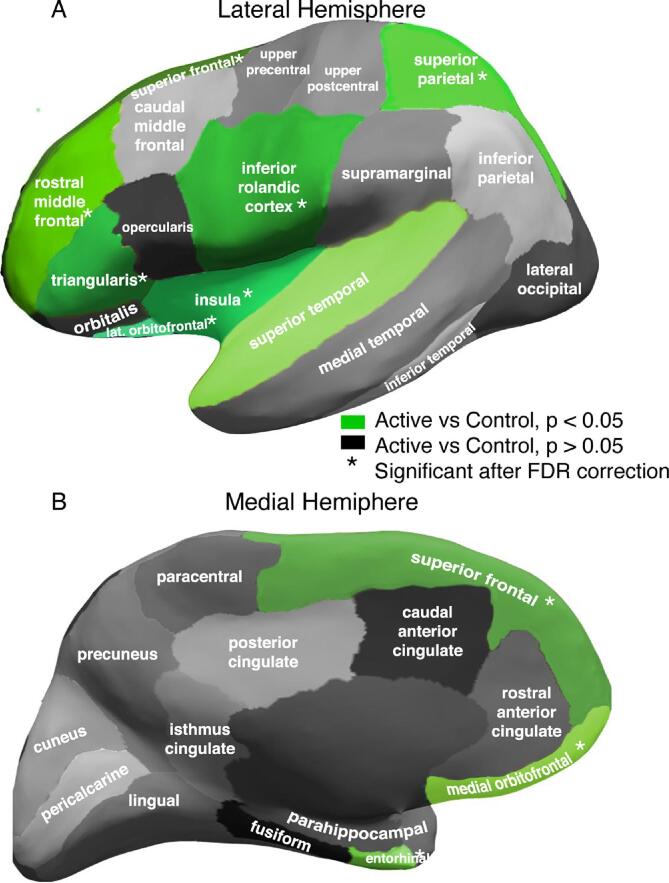
**Spindle rate deficit extends beyond inferior Rolandic cortices.** Parcellation of the cortex into 31 regions per hemisphere. Lateral **(A)** and medial **(B)** regions with a significant reduction in spindle rate in active versus control subjects indicated in green. Asterisk (*) indicates significant differences after correction for multiple comparisons. (For interpretation of the references to colour in this figure legend, the reader is referred to the web version of this article.)

To test hypothesis (2), we first (a) determined whether focal spindle rate and regional spindle rate separately predicted cognitive performance. We then (b) tested whether inclusion of the non-focal component of regional spindle rate to a model including focal spindle rate improved model performance. We define the focal spindle rate as the average over sources in the inferior Rolandic cortices. We define the regional spindle rate as the average over all sources in cortical regions identified to have a significantly lower spindle rate in subjects with active epilepsy in hypothesis (1) (see Fig. 5) and the non-focal component of regional spindle rate as the average over sources in the same cortical regions excluding the inferior Rolandic cortex.

For (2a), to evaluate the performance of each model to predict global cognitive function, we summed the log likelihood across each of the individual models (each described below), assuming independence between cognitive functions after conditioning on spindle rate. The summed log likelihood provides a measure of goodness-of-fit for each model using either the focal or regional estimates of spindle rate, *i.e.,* the focal log likelihood or the regional log likelihood respectively. We used a likelihood ratio test (4 degrees of freedom) to test if the focal or regional log likelihood significantly differed than the log likelihood of a null model of global cognitive function. If spindle rate was found to predict performance on the global model, we then analyzed the relationship between spindle rate and task performance for each individual neuropsychological model. For each task, a significant relationship was determined if *P* < 0.05.

For the individual neuropsychological tasks: (i) fine motor dexterity, (ii) processing speed, (iii) full-scale IQ, and (iv) phonological awareness, we built the following models. For (i), we paired performance by hand with spindle rate in the contralateral hemisphere (see Fig. 7A). We modeled motor dexterity as a linear mixed effects model with a random intercept to account for repeat measures from the same subject (*i.e.*, left- and right-hand performance; Equation 1). For (ii) to (iv), we fit a linear model estimating performance as a function of spindle rate (Equation 2). For (ii) and (iii), we compared task performance with the average spindle rate over the left and right hemispheres. For (iv), we compared task performance with the spindle rate in the left hemisphere, which is typically dominant in language. For all models, we applied the inverse hyperbolic sine (IHS) transform to the spindle rate to reduce the influence of extreme observations (Kramer et al., 2021). We tested age as a covariate in each model and included age as an independent variable in the model if *P* < 0.1. Doing so, we only found evidence to include age in (i); we therefore modeled motor dexterity as a function of spindle rate, controlling for age. The corresponding model for (i) fine motor dexterity is:

(1) $$\mathrm{Motor}\;\mathrm{performance}=\beta_{0}+\beta_{1}\text{IHS}\left(spindle\;rate\right)+\beta_{2}age+(1|subject),$$

where $\beta_{0},\beta_{1},\;\text{and}\;\beta_{2}$ are unknown parameters to estimate and (1|subject) indicates a random-effects term indexed by subject. For (ii) – (iv), the corresponding model is:

(2) $$\mathrm{Task}\;\mathrm{performance}=\beta_{0}+\beta_{1}\text{IHS}\left(spindle\;rate\right).$$

For (2b), we compared nested models in which the null model estimates cognitive function using focal spindle rate only (Equations 1 and 2) and the full model additionally includes the non-focal components of the regional spindle rate. In this analysis, the full model for (i) fine motor dexterity was:

(3) $$\mathrm{Motor}\;\mathrm{performance}=\beta_{0}+\beta_{1}\text{IHS}\left(spindle\;rate_{\mathrm{focal}}\right)+\beta_{2}\text{IHS}\left(spindle\;rate_{regional}\right)+\beta_{3}age+\left(1|subject\right),$$

and for (ii) – (iv), the corresponding full model is:

(4) $$\mathrm{Task}\;\mathrm{performance}=\beta_{0}+\beta_{1}\text{IHS}\left(spindle\;rate_{\mathrm{focal}}\right)+\beta_{2}\text{IHS}\left(spindle\;rate_{\mathrm{regional}}\right).$$

To test if inclusion of the non-focal components of regional spindle rate improved model performance of global cognitive function, we tested for a significant difference between the focal and regional log likelihoods using a likelihood ratio test (4 degrees of freedom). If so, we applied the same analysis to each individual neuropsychological task (likelihood ratio test, 1 degree of freedom).

To account for potential interdependencies in the neuropsychological tasks, we repeated our analysis assuming conditional dependence on IQ (see *Supplementary material*) and found qualitatively similar results.

### 2.9 Data availability

Raw data were generated at Massachusetts General Hospital and the Athinoula A. Martinos Center for Biomedical Imaging. Derived data supporting the findings of this study are available from the corresponding author on request. Software for the detection of spindle events is available at https://github.com/Mark-Kramer/Spindle-Detector-Method.

## 3 Results

### 3.1 Subject characteristics

We found no evidence of a difference in age (*P* = 0.25, one-way ANOVA) or sex (*P* = 0.07, Fisher’s exact test, Table 1) between the control subjects, active epilepsy and resolved epilepsy groups. Of the 8 children with active epilepsy, 4 were on anticonvulsant medication at the time of the EEG recording. Of the 10 children with resolved epilepsy, 6 were on anticonvulsant medication at the time of the EEG recording. Antiseizure medications (ASM) included: levetiracetam (7), lamotrigine (1), and lacosamide (1). We found no difference in the distribution of antiseizure medication status between the active and resolved epilepsy groups (*P* = 1, Fisher’s exact test, Table 1).

**Table 1:** Subject characteristics.

	Age (yrs)	Sex	ASM (%)	ASM	Duration Seizure Free (median and range)
Control	8.9–14.9	3M/5F	N/A	N/A	N/A
Active	9–14.7	5M/3F	50%	Levetiracetam (2), Lamotrigine (1), and Lacosamide (1)	54 days (10 days – 1 year)
Resolve	10.3–16.7	9M/1F	60%	Levetiracetam (5)	2.2 years (1.2 – 4.3 years)

*P*-value	0.25^†^	0.07^††^	1^††^		

ASM = antiseizure medication during EEG recording. ^†^One-way ANOVA. ^††^Fisher’s exact test.

### 3.2 Spindle rate in the inferior Rolandic cortex is reduced in active Rolandic epilepsy

We found a decrease in spindle rate in the inferior Rolandic cortices of active subjects compared to control subjects (70.9% decrease, *P =* 0.007, quasi-Poisson model) and no detectable difference in spindle rate between resolved and control subjects (*P =* 0.2; Fig. 2C; active subjects (mean ± standard deviation) 0.65 ± 0.78 spindles/min; resolved 1.09 ± 0.65 spindles/mi; control subjects 1.84 ± 1.04 spindles/min). We conclude that spindle rate is transiently decreased in the inferior Rolandic cortex in Rolandic epilepsy during the active period of disease.

We note that alternative spectral measures have been used to estimate spindle activity. In particular, sigma power is frequently used as a surrogate measure of spindle activity (Tucker and Fishbein, 2009, Nobili et al., 2001, Nobili et al., 1999, Beelke et al., 2000); although, the effects are weakened by background EEG activity (De Gennaro and Ferrara, 2003, Wamsley et al., 2012). Visual inspection of the average spectrum of source activity in the Rolandic cortices suggests lower sigma-band power in subjects with Rolandic epilepsy compared to control subjects (Fig. 3A). To examine this surrogate measure of spindle activity, we analyzed both sigma power and sigma bump, in which the background sigma activity is removed (see *Methods*). As expected (Purcell et al., 2017), both measures positively correlated with spindle rate (sigma power, *r* = 0.60, *P* < 1e-5; sigma bump, *r* = 0.82, *P* < 1e-13; Fig. 3B,C). However, we found no difference in these spectral measures between the active epilepsy and control groups (sigma power, *P* = 0.119; sigma bump, *P* = 0.08). We conclude that spindle rate is reduced in the inferior Rolandic cortices and is a more sensitive measure of the difference in spindle activity between subject groups than spectral measures alone.

### 3.3 Spindle features are typical in active Rolandic epilepsy but are less bilaterally synchronous

To test whether spindle properties in the inferior Rolandic cortices differ between subject groups, we analyzed five features from the spindles detected within the inferior Rolandic cortices (Fig. 4A-C). We found no difference between groups in four of the features: sigma power (*P* = 0.25, likelihood ratio test, see *Methods*; Fig. 4D), duration (*P* = 0.21, likelihood ratio test; Fig. 4E), or the intra-hemispheric or inter-hemispheric sigma-band coherence (*P* = 0.64, likelihood ratio test, Fig. 4F; *P* = 0.11, Fig. 4G, respectively). The interhemispheric synchrony (*e.g.,* the co-occurrence of spindles in the left and right hemispheres at the same time) was lower in active (*P* = 0.005, *t*-test of linear model coefficient), but not resolved (*P* = 0.13, *t*-test of linear model coefficient) epilepsy subjects compared to control subjects (Fig. 4H). We conclude that – although spindle rate is reduced in active Rolandic epilepsy – when spindles occur, spindle features are similar in Rolandic epilepsy and control subjects. However, spindles are more bilaterally independent in subjects with active epilepsy compared to control subjects, consistent with the bilaterally independent nature of the epileptiform spike activity in Rolandic epilepsy subjects (Carvill et al., 2013, Callenbach et al., 2010).

### 3.4 Spindle deficit extends beyond inferior Rolandic cortices

To test the hypothesis that the spindle deficit extends beyond the epileptic cortex, we analyzed spindle rates measured from each Desikan-Killiany atlas label in each subject (see *Methods*). We found significantly lower spindle rates in active epilepsy compared to control subjects (quasi-Poisson model) in frontal cortical regions (superior frontal; rostral middle frontal; triangularis; lateral orbitofrontal; medial orbitofrontal), insula, temporal cortical regions (superior temporal; entorhinal), as well as in the superior parietal region (*P* < 0.039 for all regions). After controlling for multiple comparisons using FDR, these differences remained significant in all regions (*P* < 0.009, adjusted *P* < 0.03 for all remaining regions) except the superior temporal gyrus (*P* = 0.038, adjusted *P* > 0.05; Fig. 5). The most affected region was the inferior Rolandic cortex which, as reported above, results in a mean 70.9% decrease in the baseline spindle rate for the active group. For the remaining affected regions, we find mean decreases between 49.6 and 60.8% in the baseline spindle rate for the active group (Table 2). We note that no difference in spindle rate was observed between controls and children with active Rolandic epilepsy in the superior Rolandic cortex, confirming that involvement of the Rolandic area is limited to the inferior portion. We conclude that spindle deficits in Rolandic epilepsy involve broader extra-Rolandic cortical regions beyond the inferior Rolandic cortex.

**Table 2:** Percent reduction in spindle rate by being in the active group relative to the control group.

**Brain region**	**Percent (%)**	**P-value**
Lateral Orbito. Frontal	60.8	0.001*
Rostral Middle Frontal	55	0.002*
Superior Frontal	50.4	0.006*
Medial Orbito. Frontal	56.7	0.006*
Triangularis	56.2	0.006*
Inferior Rolandic	70.9	0.007*
Superior Parietal	49.6	0.007*
Insula	51.7	0.008*
Entorhinal	58.2	0.008*
Superior Temporal	54.1	0.038
Cuneus	46.6	0.051
Pericalcarine	42	0.053
Opercularis	46.2	0.056
Caudal Middle Frontal	57.4	0.063
Rostral Ant. Cingulate	61.5	0.074
Inferior Parietal	58.5	0.075
Fusiform	49.5	0.103
Caudal Ant. Cingulate	47.8	0.103
Lateral Occipital	52.9	0.123
Inferior Temporal	33.5	0.125
Lingual	31.3	0.135
Orbitalis	43.9	0.14
Medial Temporal	48.2	0.157
Precentral Gyrus (excluding inferior Rolandic cortex)	42.6	0.202
Precuneus	43.8	0.21
Posterior Cingulate	39.4	0.225
Supramarginal	41	0.249
Isthmus Cingulate	18	0.68
Parahippocampal	8.6	0.783
Paracentral	4.7	0.877
Postcentral Gyrus (excluding inferior Rolandic cortex)	−3.2	0.92

Asterisk (*) indicates significant differences after correcting for multiple

comparisons using the false discovery rate.

### 3.5 Regional estimates of spindle rate predict cognitive function better than focal estimates

We found that focal source estimates of spindle rate from the inferior Rolandic cortex only, and regional estimates of spindle rate from all affected cortical regions, both predicted global cognitive performance (focal *P* = 0.002, regional *P* < 1e-4, focal log likelihood = -156.7, regional log likelihood = -153, null log likelihood = -165.5, likelihood ratio test, 4 degrees of freedom). Adding the regional component of spindle rate to a model with only the focal component trended to improve model performance (*P* = 0.052, regional log likelihood = -152, likelihood ratio test, 4 degrees of freedom).

**Fig. 6:**
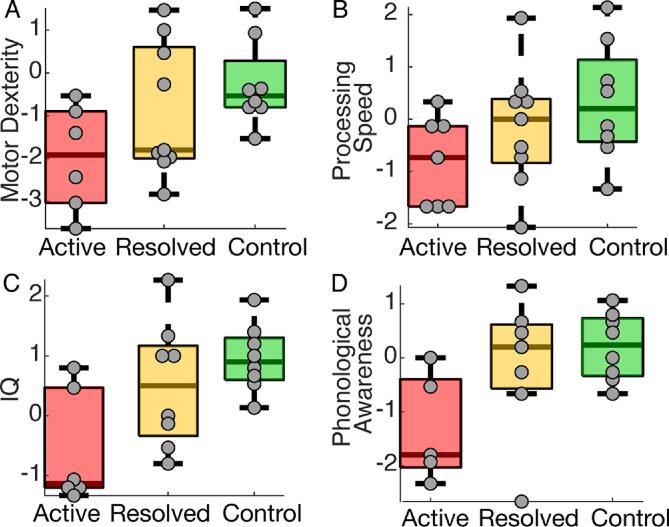
**Performance on neuropsychological assessments.** Each subject’s performance (z-score, circles) on the (A) motor dexterity (dominant and nondominant scores averaged per subject), (B) processing speed, (C) IQ, and (D) phonological awareness tasks. In each boxplot, central mark indicates the median; bottom and top edges of the box indicate the 25th and 75th percentiles, respectively; and whiskers extend to the most extreme data points.

**Fig. 7:**
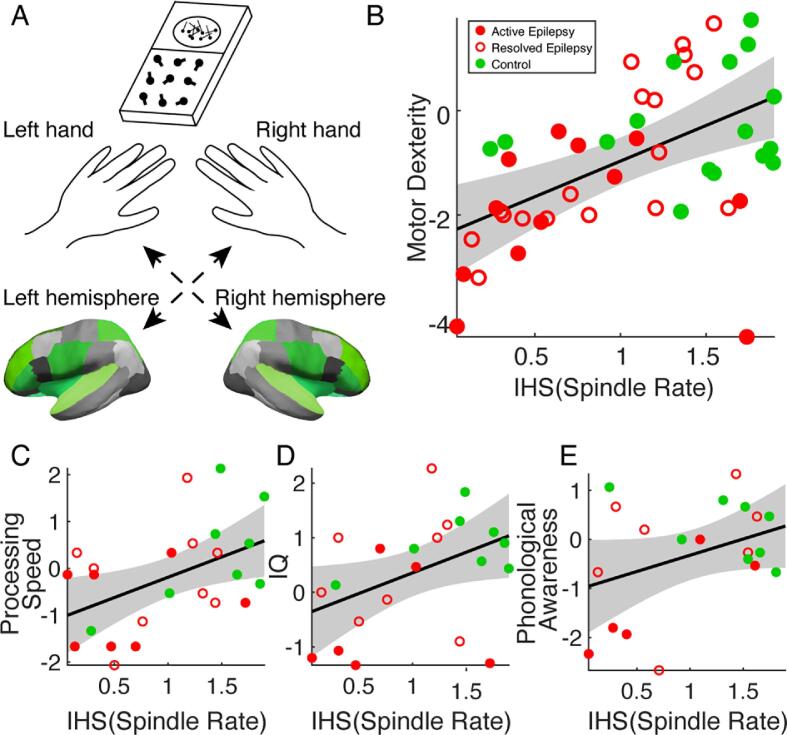
**Regional spindle rate correlates with neuropsychological assessments.** (**A**) Schematic of the grooved pegboard experiment. Subjects perform a grooved pegboard task with their left and right hand. Performance is paired with spindle rate in the contralateral hemisphere (green). **(B-D)** As regional measure of spindle rate increases, motor performance (**B**), processing speed (**C**), and IQ (**D**) significantly increase. (**E**) Phonological awareness shows an increasing trend. Circles represent three disease groups: active (red filled), resolved (red unfilled), and control subjects (green). The solid line indicates the model fit, and shaded regions indicate 95% confidence intervals. (For interpretation of the references to colour in this figure legend, the reader is referred to the web version of this article.)

Across individual neuropsychological tasks (distributions shown in Fig. 6), we found positive relationships between both focal and regional source estimates of spindle rate and performance in each domain tested: fine motor skills, processing speed, full-scale IQ, and phonological awareness (Fig. 7). Using the focal spindle estimates, we found strong positive relationships between spindle rate and fine motor performance ($\beta_{1}=$ 0.9, see *Methods*, 95% CI [0.15,1.65], *P* = 0.02, R^2^ = 0.88), and processing speed ($\beta_{1}=$ 0.84, 95% CI [0.11, 1.57], *P* = 0.03, R^2^ = 0.19), and weaker positive relationships with full-scale IQ ($\beta_{1}=$ 0.76, 95% CI [0.04, 1.5], *P* = 0.052), and phonological awareness ($\beta_{1}=$ 0.79, 95% CI [0.005, 1.58], *P* = 0.06). Using the regional spindle estimates (Fig. 7), we found strong positive relationships between spindle rate and motor performance ($\beta_{1}=$ 1.36, 95% CI [0.63, 2.1], *P* < 1e-3, R^2^ = 0.9), processing speed ($\beta_{1}=$ 0.87, 95% CI [0.19, 1.54], *P* = 0.02, R^2^ = 0.22), and IQ ($\beta_{1}=$ 0.76, 95% CI [0.07, 1.44], *P* = 0.04, R^2^ = 0.19), and no relationship with phonological awareness (*P* = 0.1); see Table 3.

**Table 3:** Model fits for each neuropsychological assessment using focal and regional spindle rate.

	**(i) Fine Motor Skills**	**(ii) Processing Speed**	**(iii) Full-Scale IQ**	**(iv) Phonological Awareness**
**Focal Spindle Rate**				
Beta Coefficient (95% CI)	0.9 (0.15,1.65)	0.84 (0.11, 1.57)	0.76 (0.04, 1.5)	0.79 (0.005, 1.58)
P-value	0.02	0.03	0.052	0.06
Log likelihood	−65.1	–33.7	−29.6	−28.3
R^2^	0.88	0.19	0.18	0.18
**Regional Spindle Rate**				
Beta Coefficient (95% CI)	1.36 (0.63, 2.1)	0.87 (0.19, 1.54)	0.76 (0.07,1.44)	0.66 (-0.08, 1.41)
P-value	<1e-3	0.02	0.04	0.1
Log likelihood	−61.7	–33.2	−29.4	−28.7
R^2^	0.9	0.22	0.19	0.14

Across the individual tasks, adding the regional spindle rate to a model with only the focal component significantly improved model performance to predict fine motor skills (*P* = 0.006, focal log likelihood = -65.1, regional log likelihood = -61.2, likelihood ratio test, 1 degree of freedom). We found no significant difference between models using focal only or combined focal and regional spindle estimates to predict processing speed, full-scale IQ, and phonological awareness (*P* > 0.3).

We note that repeating these analyses without removing interictal spikes from the data (see *Artifact and epileptic spike removal procedures*) yielded qualitatively consistent results. In addition, including sex or medication status in the model as additional predictors yielded consistent results. We also note that the model residuals of IQ are correlated with the model residuals of motor dexterity, processing speed, and phonological awareness (Pearson’s correlation coefficient *r* > 0.53, *P* < 0.012 for all models). To account for this, we repeated our analysis assuming conditional dependence on IQ (see *Supplementary material*) which removed all correlation between the models (*r* < 0.18, *P* > 0.12) and found qualitatively similar results.

We conclude that both focal and regional estimates of spindle rate predict cognitive function. However, regional estimates of spindle rate from all affected cortical regions trended to improve prediction of global cognitive function and significantly improved prediction of motor performance compared to spindle estimates limited to the inferior Rolandic cortex.

## 4 Discussion

While neurocognitive deficits commonly occur in Rolandic epilepsy, it is unknown how the pathology of epilepsy disrupts cognition in this disease and related epileptic encephalopathies. Here, using ESI, we investigated the spatial extent of the sleep spindle deficit and the relationships between cortical sleep spindle deficits and performance on neurocognitive tasks. We found that children with active Rolandic epilepsy have regional spindle deficits that extend beyond the epileptic Rolandic cortices, involving parts of the pre-frontal, insula, temporal, and parietal cortices. We also found that inclusion of regional spindle rates estimated from these broadly affected regions better predicted cognitive performance on a range of tasks compared to spindle rate estimated from the inferior Rolandic cortex alone. These results suggest that the cognitive symptoms in Rolandic epilepsy might be due to involvement of broader regional networks beyond the Rolandic cortex and contributes to growing evidence of thalamocortical circuit dysfunction in Rolandic epilepsy.

Sleep spindles - discrete bursts of 10–15 Hz oscillations during NREM sleep - are standard features of NREM sleep and linked to general measures of intelligence (Beenhakker and Huguenard, 2009). Sleep spindles have been causally linked to sleep dependent memory consolidation in animal work (Fogel and Smith, 2011, Westermann et al., 2015, Fernandez and Lüthi, 2020, Latchoumane et al., 2017). We have recently identified spindle deficits in sleep-activated developmental epileptic encephalopathies (Kramer et al., 2021, Stoyell et al., 2021). As sleep spindles originate in the thalamic reticular nucleus (TRN) and are propagated in thalamocortical feedback circuits (De Gennaro and Ferrara, 2003, Beenhakker and Huguenard, 2009), their disruption localizes pathology to the thalamocortical circuit. Here we identify a regional spindle deficit in Rolandic epilepsy, which could have mechanistic and treatment implications for the broad range of neurobehavioral symptoms affecting these children that extend beyond the Rolandic cortex and in related developmental epileptic encephalopathies with thalamocortical circuit dysfunction.

The identification of a regional spindle deficits suggests two potential sources of malfunctioning in the thalamocortical circuitry. First, the thalamus is comprised of many nuclei that have broad and discrete thalamocortical connectivity (Behrens et al., 2003, Fama and Sullivan, 2015, Bastuji et al., 2020). The TRN is comprised of GABAergic cells (Clemente-Perez et al., 2017) and has bidirectional communications with other thalamic nuclei (Zikopoulos and Barbas, 2006). Both the thalamic nuclei (Behrens et al., 2003) and GABAergic subpopulations (Clemente-Perez et al., 2017, Li et al., 2020) have discrete thalamocortical circuitry potentially leading to the spatially discrete regional disturbances in cortical spindle activity (Bastuji et al., 2020). For example, a focal reduction of spindle activity in the inferior Rolandic cortices could implicate ventroanterior, ventrolateral, and ventroposterior thalamic nuclei (Andersen et al., 1967). However, regional spindle deficits involving the prefrontal, superior parietal, insular, and temporal regions could implicate more thalamic nuclei, including the anterior pulvinar, mediodorsal nucleus and parts of the anterior nucleus (Behrens et al., 2003). Second, it has been shown in mouse models that parvalbumin (PV) and somatostatin (SOM) cells in the TRN are part of distinct functional circuits. For example, PV cells have strong inputs to thalamic relay nuclei (*e.g.*, ventromedial, ventrolateral, ventroposteriormedial, ventroposteriorlateral) whereas SOM cells have strong inputs to intralaminar nuclei (Clemente-Perez et al., 2017). Although sleep spindles enable identification of cortical networks affected, future work is required to understand the regional thalamocortical circuitry leading to the distributed cortical abnormalities observed.

We analyzed alternative measures of spindle activity, sigma power and sigma bump. Despite correlation with spindle rate, sigma power and sigma bump (De Gennaro and Ferrara, 2003, Nobili et al., 2001, Beelke et al., 2000) were not as sensitive as spindle rate alone to detect a difference between groups. While we found no significant difference in spindle rate between the resolved and control groups, we note that the mean spindle rate for the resolved group lies between the active and control groups. This suggests there may be residual disease in these children or that a subset of children classified as resolved using our definition may not have achieved complete disease resolution. Sigma power is frequently used as an approximation of spindle rate (Goldstone et al., 2019) and changes in spindle parameters have been identified in other developmental disorders (*e.g.,* Farmer et al., 2018 (Farmer et al., 2018), Gruber et al., 2016 (Gruber and Wise, 2016), and Shibagaki et al., 1982 (Shibagaki et al., 1982). Here, we found no differences in any spindle power or duration between the active epilepsy and control subjects. We also found no difference in the fine temporal coupling (*i.e.,* sigma coherence) between spindles; however, the synchrony of spindles between homologous regions across hemispheres was reduced in active Rolandic epilepsy compared to controls. This finding indicates that spindle production is more bilaterally independent in active Rolandic epilepsy, similar to the bilaterally independent epileptic spiking activity (Galicchio et al., October 2020). Altogether, these results suggest that the spindle pathology and the associated neural plastic changes that contribute to cognitive deficits in Rolandic epilepsy are restricted to the spindle rate, and not characteristics or coupling properties of the spindles themselves. Further, these data suggest that the process resulting in a reduction of spindles occurs independently in the left and right thalamocortical circuits.

In analyzing neurocognitive task performance, we found a strong relationship between the regional spindle rate and motor dexterity. Source estimates of regional spindle rate improved prediction of contralateral fine motor performance compared to focal estimates of spindle rate from the inferior Rolandic estimates, where regional estimates explained 90% of the variance in fine motor performance and increased the mean coefficient estimate $\left(\beta_{1}\right)$ by approximately 50% compared to the focal spindle rate. Although the inferior Rolandic cortex is involved in primary sensorimotor processing, several regions identified to have a spindle deficit, including the posterior parietal cortex and pre-frontal cortex, are involved in the planning, initiation and execution of motor movements (Purves et al., 2004, Andersen et al., 2019). Additionally, the insula has strong functional connectivity with the inferior Rolandic cortex (Fink et al., 1997). Thus, the fine motor impairments measured in this task may reflect dyscoordination in these distributed motor networks that extend beyond the primary Rolandic cortex. Although we did not find that the regional model significantly improved performance to predict processing speed and full-scale IQ compared to the focal model, we note increases in both R^2^ and mean coefficient estimates $\left(\beta_{1}\right)$ in models using regional spindle rate, suggesting nominal improvements. We also note that since we only had one measurement per subject for these tests, we may not have had as much power to detect a difference as we did when analyzing motor performance, where we had both left and right hand measurements. Thus, distributed cortical networks may also contribute to these cognitive deficits observed. To limit risk of false detections due to multiple comparisons, we did not test spindle estimates from mixed combinations of cortical regions here. However, given the regional spindle deficits observed here, future work could investigate whether spindle estimates from different combinations of cortical regions improve models of specific cognitive functions.

Our study was limited by small sample sizes and while this suggests that a large effect is present, replication in another cohort would support the generalizability of these findings. A potential confound is higher spindle rates could be due to more sustained stage 2 NREM sleep (Purcell et al., 2017) though each of our subjects was provided only a short nap opportunity, and it has been reported that even shorter naps can significantly improve memory retention (Diekelmann and Born, 2010). Further, although EEG is sensitive to both radially and tangentially oriented cortical sources, EEG and subsequently EEG source imaging has reduced sensitivity to detect activity in deep cortical sources, such as the orbitofrontal cortex or the insula (Puce and Hämäläinen, 2017). However, this limitation would be expected to impact each group similarly. Finally, we did not have the power to detect a subtle impact of antiseizure medications on cognitive function and this should be evaluated in future work.

Here, we have provided evidence that spindle rate is a sensitive biomarker that tracks with disease state and extends beyond the region of focal spiking activity, implicating regional thalamocortical circuit dysfunction. Although Rolandic epilepsy is considered a focal epilepsy, we found the regional model of dysfunction better predicts cognitive function, providing a potential mechanistic explanation for the range of cognitive deficits observed in children with this epileptic encephalopathy. Alongside treating seizures, future therapeutic trials in Rolandic epilepsy could target increased spindle production with the goal of improving cognitive symptoms in this common disease (Mednick et al., 2013, Ngo et al., 2015).
